# A deep learning-based algorithm for pulmonary tuberculosis detection in chest radiography

**DOI:** 10.1038/s41598-024-65703-z

**Published:** 2024-06-28

**Authors:** Chiu-Fan Chen, Chun-Hsiang Hsu, You-Cheng Jiang, Wen-Ren Lin, Wei-Cheng Hong, I.-Yuan Chen, Min-Hsi Lin, Kuo-An Chu, Chao-Hsien Lee, David Lin Lee, Po-Fan Chen

**Affiliations:** 1 Division of Chest Medicine, Department of Internal Medicine, Kaohsiung Veterans General Hospital, Kaohsiung, Taiwan, R.O.C.; 2 Shu-Zen Junior College of Medicine and Management, Kaohsiung, Taiwan, R.O.C.; 3 Department of Nursing, Mei-Ho University, Pingtung, Taiwan, R.O.C.; 4 Department of Obstetrics and Gynecology, National Cheng Kung University Hospital, College of Medicine, National Cheng Kung University, Tainan, Taiwan, R.O.C.; 5 Quality Center, National Cheng Kung University Hospital, College of Medicine, National Cheng Kung University, Tainan, Taiwan, R.O.C.

**Keywords:** Artificial intelligence, Chest X-ray, Deep learning, Neural network, Tuberculosis, Tuberculosis, Machine learning

## Abstract

In tuberculosis (TB), chest radiography (CXR) patterns are highly variable, mimicking pneumonia and many other diseases. This study aims to evaluate the efficacy of Google teachable machine, a deep neural network-based image classification tool, to develop algorithm for predicting TB probability of CXRs. The training dataset included 348 TB CXRs and 3806 normal CXRs for training TB detection. We also collected 1150 abnormal CXRs and 627 normal CXRs for training abnormality detection. For external validation, we collected 250 CXRs from our hospital. We also compared the accuracy of the algorithm to five pulmonologists and radiological reports. In external validation, the AI algorithm showed areas under the curve (AUC) of 0.951 and 0.975 in validation dataset 1 and 2. The accuracy of the pulmonologists on validation dataset 2 showed AUC range of 0.936–0.995. When abnormal CXRs other than TB were added, AUC decreased in both human readers (0.843–0.888) and AI algorithm (0.828). When combine human readers with AI algorithm, the AUC further increased to 0.862–0.885. The TB CXR AI algorithm developed by using Google teachable machine in this study is effective, with the accuracy close to experienced clinical physicians, and may be helpful for detecting tuberculosis by CXR.

## Introduction

Tuberculosis (TB) is one of the most important infectious diseases worldwide and causes millions of illnesses and deaths annually^1^. Chest radiography is an essential first-line diagnostic tool for TB because of its low cost and speed. However, the characteristics of TB chest X-ray (CXR) are highly variable, mimicking pneumonia and many other diseases. The atypical pattern is particularly common in elderly patients, immunocompromised, and those with multiple comorbidities^2,3^. Consequently, the early diagnosis of TB using CXRs can be challenging. Moreover, CXR reports often cannot be completed in a timely manner, this also increases the difficulty of early TB detection for the frontline clinicians.

The application of artificial intelligence (AI) to CXR for TB is a field with tremendous potential. The deep neural network-based image interpretation has achieved remarkable results in the field of medical imaging. Recent research has developed numerous medical image recognition algorithms for CXR patterns^4–6^ and various pulmonary diseases (pneumonia, lung cancer, TB, pneumothorax, COVID-19, etc.)^7–15^, in some of them the accuracy can match or even outperform that of radiologists. Some of them had external validation confirmed accuracy^7–10,12,14,16,17^. In a study evaluation CXR algorithms for pulmonary diseases classification, the combination of algorithm with physicians successfully improve accuracy than physicians alone. And the benefit is observed in both radiologists and non-radiology physicians^8^. In another study evaluating CXR algorithm for TB detection, a similar accuracy benefit is found in physicians with algorithm assistance^7^. Five commercial TB AI algorithms had been carefully validated, and the specificity ranged from 61 to 74% when sensitivity was fixed at 90%^13,14^.

In 2019, Google Teachable Machine (GoogleTM) launched its second version^18^. This tool allows users to train deep neural networks for image recognition using a graphical user interface on a Chrome browser with almost no coding required. Its highly lightweight design, along with its use of transfer learning techniques, significantly reduces the computational requirements and amount of data required for training. This implies that AI training can be simply performed using a desktop or laptop computer. Therefore, the purpose of this study is to assess the feasibility and accuracy of GoogleTM, for the detection of CXR images in patients with TB. To see its utility in clinical practice, we also plan to compare the accuracy of this simple AI tool to the frontline physicians.

## Materials and methods

This study was designed to use freely available open TB CXR datasets as training data for our AI algorithm. Subsequent accuracy analyses were performed using independent CXR datasets and actual TB cases from our hospital. All image data were de-identified to ensure privacy. This study was reviewed and approved by institutional review board (IRB) of Kaohsiung Veterans General Hospital, which waived the requirement for informed consent (IRB no.: KSVGH23-CT4-13). This study adheres to the principles of the Declaration of Helsinki.

### Training datasets

**Figure 1:**
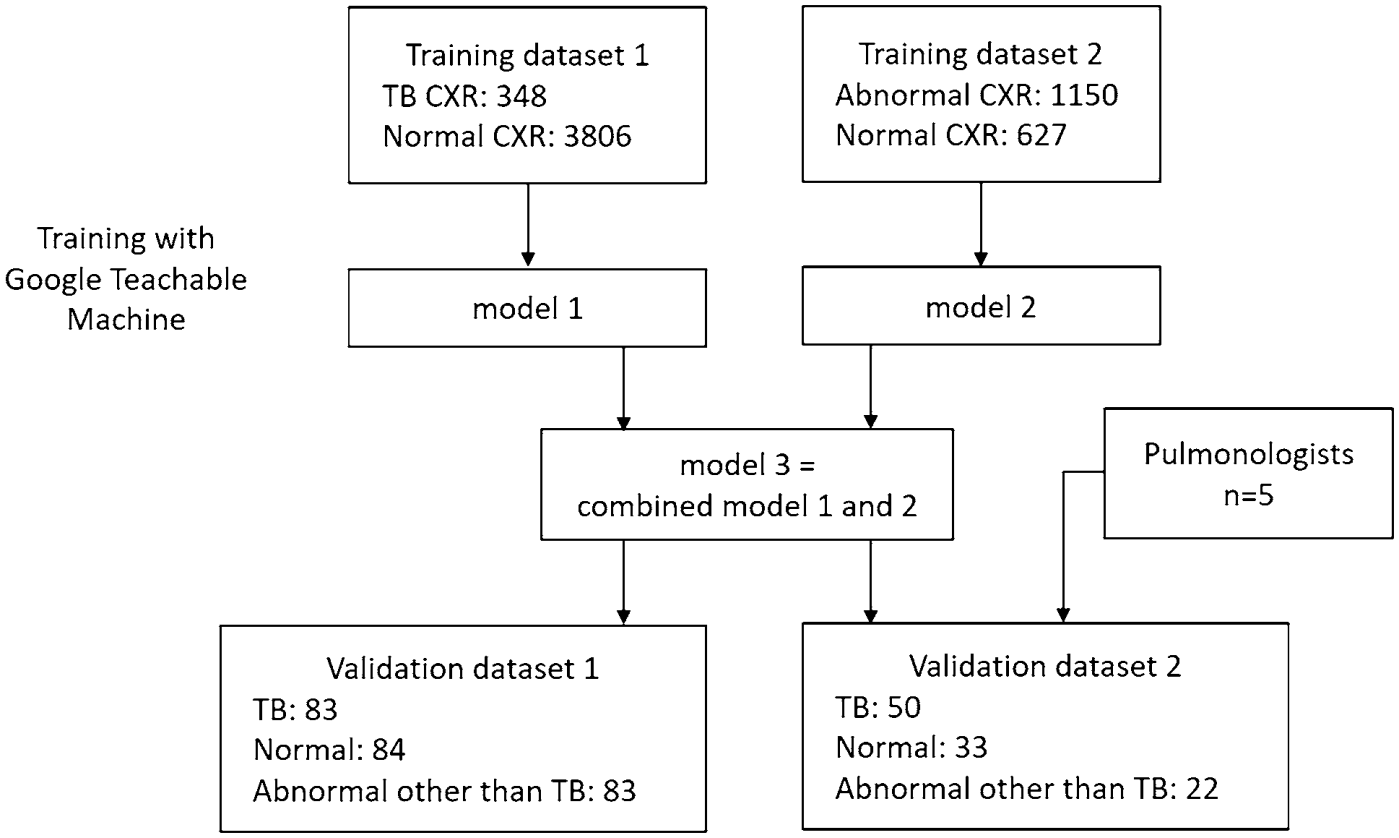
Flow chart of model training and validations.

The flowchart of the study design is shown in Fig. 1. Due to a high prevalence of TB and varied imaging presentation, TB cannot be entirely excluded in case of CXR presenting with pneumonia or other entities. Our preliminary research indicated that training a model solely on TB vs. normal resulted in bimodally distributed predictive values. Therefore, CXRs that were abnormal but not indicative of TB usually had predictive value too high or too low, and failed to effectively differentiate abnormal cases from normal or TB. For common CXR abnormalities such as pneumonia and pleural effusion, the TB risk is lower, but not zero. Thus, we trained two models using 2 different training datasets, one for TB detection and another for abnormality detection. Then the output predictive values were averaged.

The features of the CXR datasets for training is summarized in Table 1. The inclusion criteria are CXR of TB, other abnormality, or normal. Both posteroanterior view and anteroposterior view CXRs are included. The exclusion criteria are CXR with poor quality, lateral view CXR, children CXR, and those with lesions too small to detect at 224 × 224 pixels size). All the CXR images were confirmed by C.F.C. to ensure both image quality and correctness.

**Table 1:** Summary of datasets and chest X-rays used in this study.

	n	Age, years	Male (%)
Training dataset 1			
TB	348		
Shenzhen dataset	301	34 (27–45)	213 (68.5)
Montgomery dataset	47	47 (33–61)	32 (68.1)
Normal	3806		
Shenzhen dataset	287	31 (25–40)	201 (67.7)
Montgomery dataset	75	34 (23–47)	24 (32)
NLM and RNSA dataset	3444	NA	NA
Training dataset 2 (Chest X-ray 14 dataset)			
Abnormal	1150	NA	NA
Cardiomegaly	235		
Consolidation	185		
Pulmonary edema	139		
Pleural effusion	230		
Mass	255		
Pulmonary fibrosis	106		
Normal	627	NA	NA
Validation dataset 1			
TB	83	74 (62–88)	69 (83.1)
Normal	84	49 (37–62)	36 (42.8)
Abnormality other than TB	83	71 (64–86)	68 (81.9)
Pneumonia	73		
Pleural effusion	14		
Heart failure	10		
Fibrosis	4		
Validation dataset 2			
TB	50	74.5 (63–89)	43 (86)
Normal	33	48 (35–66)	17 (51.5)
Abnormality other than TB	22	69 (64–86)	18 (81.8)
Pneumonia	22		
Pleural effusion	5		
Heart failure	1		
Fibrosis	1		

Data is presented as median (IQR).

*NA*  not available.

Training dataset 1 is used for training algorithms to detect typical TB pattern on CXR. 348 TB CXRs and 3806 normal CXRs were collected from various open datasets for training, including the Shenzhen dataset from Shenzhen No. 3 People’s Hospital, the Montgomery dataset^19,20^, and Kaggle's RSNA Pneumonia Detection Challenge^21,22^.

Training dataset 2 is used for training algorithms to detect CXR abnormalities. A total of 1150 abnormal CXRs and 627 normal CXRs were collected from the ChestX-ray14 dataset^23^. The abnormal CXRs consisted of consolidation: 185, cardiomegaly: 235, pulmonary edema 139, pleural effusion: 230, pulmonary fibrosis 106, and mass: 255.

### Algorithm: Google teachable machine

In this study, we employed GoogleTM^18^, a free online AI software dedicated to image classification. GoogleTM provides a user-friendly web-based graphical interface that allows users to execute deep neural network computations and train image classification models with minimal coding requirements. By utilizing the power of transfer learning, GoogleTM significantly reduces the computational time and data amount required for deep neural network training. Within GoogleTM, the base model for transfer learning was MobileNet, a model pretrained by Google on the ImageNet dataset featuring 14 million images and capable of recognizing 1,000 classes of images. Transfer learning is achieved by modifying the last 2 layers of the pre-trained MobileNet, and then keep subsequent specific image recognition training^18,24^. In GoogleTM , all images are adjusted and cropped to 224 × 224 pixels for training. 85% of the image is automatically divided into training dataset, and the remaining 15% into validation dataset to calculate the accuracy.

The hardware employed in this study included a 12th-generation Intel Core i9-12900K CPU with 16 cores, operating at 3.2–5.2 GHz, an NVIDIA RTX A5000 GPU equipped with 24GB of error-correction code (ECC) graphics memory, 128 GB of random-access memory (RAM), and a 4TB solid-state disk (SSD).

### Dataset for external validation

To evaluate the accuracy of the algorithms, we collected clinical CXR data for TB, normal cases, and pneumonia/other disease from our hospital.

Validation dataset 1 included 250 de-identified CXRs retrospectively collected from VGHKS. The CXRs dates were between January 1, 2010 and February 27, 2023. This dataset included 83 TB (81 confirmed by microbiology, and 2 confirmed by pathology), 84 normal, and 83 abnormal other than TB cases (73 pneumonia, 14 pleural effusion, 10 heart failure, and 4 fibrosis. Some cases had combined features). The image size of these CXRs ranged from width: 1760–4280 pixels and height: 1931–4280 pixels.

Validation dataset 2 is a smaller dataset derived from validation dataset 1, for comparison of algorithm and physician’s performance, and included 50 TB, 33 normal and 22 abnormal other than TB cases (22 pneumonia, 5 pleural effusion, 1 heart failure, and 1 fibrosis) CXRs. The features of the two validation datasets are provided in Table 1.

Data collected from clinical CXR cases included demographic data (such as age and sex), radiology reports, clinical diagnoses, microbiological reports, and pathology reports. All clinical TB cases included in the study had their diagnosis confirmed by microbiology or pathology. Their CXR was performed within 1 month of TB diagnosis. Normal CXRs were also reviewed by C.F.C. and radiology reports were considered. Pneumonia/other disease cases were identified by reviewing medical records and examinations, with diagnoses made by clinical physicians’ judgement, and without evidence of TB detected within three months period.

### Physician’s performance test

We employed validation dataset 2 to evaluate the accuracy of TB detection of 5 clinical physicians (five board-certified pulmonologists, average experience 10 years, range 5–16 years). Each physician performed the test without additional clinical information, and was asked to estimate the probability of TB in each CXR, consider whether sputum TB examinations were needed, and make a classification from three categories: typical TB pattern, normal pattern, or abnormal pattern (less like TB).

We also collected radiology reports from validation dataset 2 to evaluate their sensitivity for detecting TB. Reports mentioning suspicion of TB or mycobacterial infection were classified as typical TB pattern. Reports indicating abnormal patterns such as infiltration, opacity, pneumonia, effusion, edema, mass, or tumor (but without mentioning “tuberculosis”, “TB”, or “mycobacterial infection”) were classified as abnormal pattern (less like TB). Reports demonstrating no evident abnormalities were classified as normal pattern. Furthermore, by analyzing the pulmonologists’ decisions regarding sputum TB examinations, we estimate the sensitivity of TB detection in pulmonologist’s actual clinical practice.

### Statistical analysis

Continuous variables are represented as mean ± standard deviation (SD) or median (interquartile range [IQR]), while categorical variables are represented as number (percentage). For accuracy analysis, the receiver operating characteristic (ROC) curve was used to compute the area under the curve (AUC). Sensitivity, specificity, positive predictive value (PPV), negative predictive value (NPV), likelihood ratio (LR), overall accuracy, and F1 score were calculated. A confusion matrix was used to illustrate the accuracy of each AI model. Boxplots were used to evaluate the distribution of the predicted values of the AI models for each etiology subgroup.

The formulas for each accuracy calculation are as follows:

(TP is true positives, TN is true negatives, FP is false positives, FN is false negatives, P is all positives, and N is all negatives.)

$$\begin{gathered} {\text{P }} = {\text{ TP}} + {\text{FN}}, \hfill \\ {\text{N }} = {\text{ TN}} + {\text{FP}}, \hfill \\ {\text{Sensitivity }} = {\text{ TP}}/{\text{P }} \times {1}00, \hfill \\ {\text{Specificity }} = {\text{ TN}}/{\text{N }} \times {1}00, \hfill \\ {\text{PPV }} = {\text{ TP}}/\left( {{\text{TP}} + {\text{FP}}} \right) \, \times {1}00, \hfill \\ {\text{NPV }} = {\text{ TN}}/\left( {{\text{TN}} + {\text{FN}}} \right) \, \times {1}00, \hfill \\ {\text{LR}} + \, = {\text{ sensitivity}}/\left( {{1} - {\text{specificity}}} \right), \hfill \\ {\text{LR}} - \, = \, \left( {{1} - {\text{sensitivity}}} \right)/{\text{specificity}}, \hfill \\ {\text{Overall accuracy }} = \, \left( {{\text{TP }} + {\text{ TN}}} \right)/\left( {{\text{P}} + {\text{N}}} \right) \, \times {1}00, \hfill \\ {\text{F1 score }} = \, \left( {{2 } \times {\text{ sensitivity }} \times {\text{ PPV}}} \right)/\left( {{\text{sensitivity }} + {\text{ PPV}}} \right) \, \times {1}00, \hfill \\ \end{gathered}$$

## Results

In this study, model 1 was trained by training dataset 1 (TB vs. normal), with the purpose to detect typical TB pattern on CXR. Model 2 was trained by training dataset 2 (abnormal vs. normal), with the purpose to detect CXR abnormalities. Each training dataset was trained at least 10 times, and the algorithm with the best overall accuracy was chosen. In model 2, twofold data augmentation was performed by zoom in method. Model 3 was a combination of model 1 and model 2, by averaging the predictive values of the two models. It was developed to detect both TB and other CXR abnormalities.

### Internal validation

The internal validation results calculated during training showed excellent accuracy: model 1 showed a sensitivity of 0.96, specificity of 0.98, and overall accuracy of 0.97. Model 2 exhibited a sensitivity of 0.92, specificity of 0.92, and an overall accuracy of 0.92. A detailed analysis of the accuracy is provided in Table 2, and the confusion matrix is provided in Table S1. The hyperparameters in training GoogleTM, and the accuracy curve and loss function were shown in Figure S1 and S2.

**Table 2:** Accuracy of Google teachable machine algorithms (internal validation).

	Sensitivity	Specificity	PPV	NPV	LR +	LR−	OA	F1 score
Model 1	0.96	0.98	0.81	0.99	48	0.04	0.97	0.88
Model 2	0.92	0.92	0.95	0.85	11.5	0.08	0.92	0.93

*LR*  likelihood ratio, *NPV*  negative predictive value, *OA*  overall accuracy, *PPV*  positive predictive value.

### External validation

**Figure 2:**
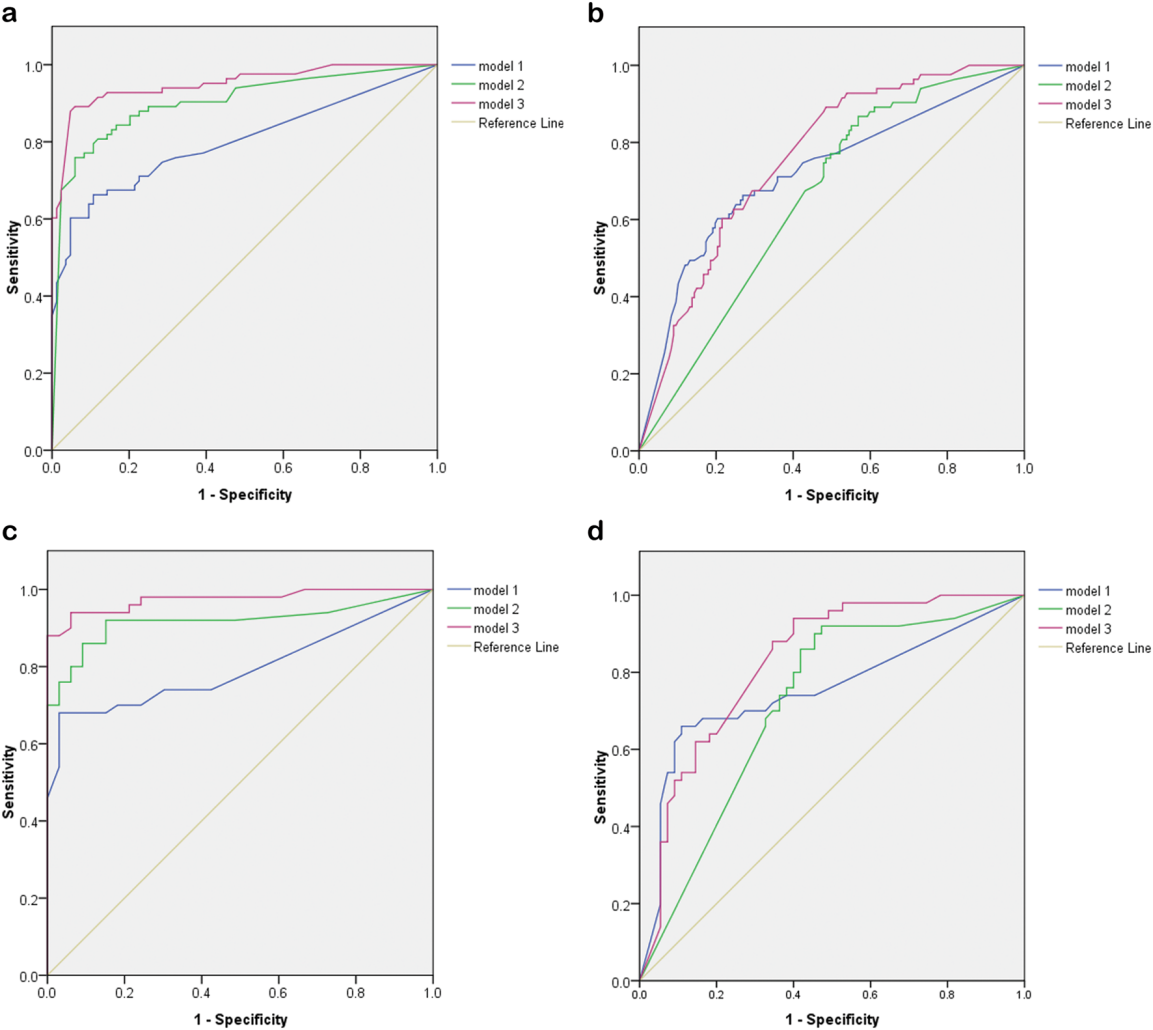
Receiver operating characteristic curves of AI models in external validations. (**a**) Validation dataset 1: TB vs. normal, (**b**) validation dataset 1: TB vs. normal and abnormal other than TB, (**c**) validation dataset 2: TB vs normal, (**d**) validation dataset 2: TB vs. normal and abnormal other than TB. *TB* tuberculosis, *AI* artificial intelligence.

The accuracy analysis for external validation is shown in Table 3 and Fig. 2a–d. For the analysis of TB vs. normal, model 1 showed AUC of 0.8 and 0.795 in validation dataset 1 and 2, respectively. Model 2 showed AUC of 0.902 and 0.917. Model 3 demonstrated better accuracy, with AUC of 0.951 and 0.975, respectively. For the analysis of TB vs. normal and abnormal other than TB, model 1 showed AUC of 0.72 and 0.752 in validation dataset 1 and 2, respectively. Model 2 showed AUC of 0.656 and 0.718. Model 3 showed AUC of 0.758 and 0.828.

**Table 3:** Accuracy of Google teachable machine algorithms (external validation; cutoff level = 0.5).

	AUC	Sen	Sp	PPV	NPV	LR+	LR−	OA	F1 score
Validation dataset 1 (TB vs. normal)									
Model 1	0.800	0.65	0.89	0.85	0.72	5.90	0.39	0.77	0.73
Model 2	0.902	0.83	0.83	0.82	0.83	4.88	0.20	0.83	0.82
Model 3	0.951	0.88	0.95	0.94	0.88	17.6	0.12	0.91	0.91
Validation dataset 1 (TB vs. normal and other abnormality)									
Model 1	0.720	0.65	0.73	0.54	0.80	2.40	0.47	0.70	0.59
Model 2	0.656	0.83	0.44	0.42	0.83	1.48	0.38	0.56	0.56
Model 3	0.758	0.88	0.52	0.47	0.89	1.83	0.23	0.64	0.61
Validation dataset 2 (TB vs. normal)									
Model 1	0.795	0.68	0.93	0.93	0.65	9.71	0.34	0.78	0.78
Model 2	0.917	0.86	0.90	0.92	0.81	8.60	0.15	0.87	0.89
Model 3	0.975	0.86	1.0	1.0	0.82	Infinity	0.14	0.91	0.92
Validation dataset 2 (TB vs. normal and other abnormality)									
Model 1	0.752	0.68	0.81	0.76	0.73	3.57	0.39	0.74	0.72
Model 2	0.718	0.86	0.58	0.65	0.82	2.04	0.24	0.71	0.74
Model 3	0.828	0.86	0.65	0.69	0.83	2.45	0.21	0.75	0.76

*AUC* area under curve, *LR* likelihood ratio, *NPV* negative predictive value, *OA* overall accuracy, *PPV* positive predictive value, *Sen* sensitivity, *Sp* specificity.

Both datasets revealed that model 3 outperformed model 1 and 2, with the best AUC, overall accuracy and F1 score. The distribution of predictive values of model 1 to model 3 in each disease subgroup were provided in Figure S3.

### Physicians’ performance

**Figure 3:**
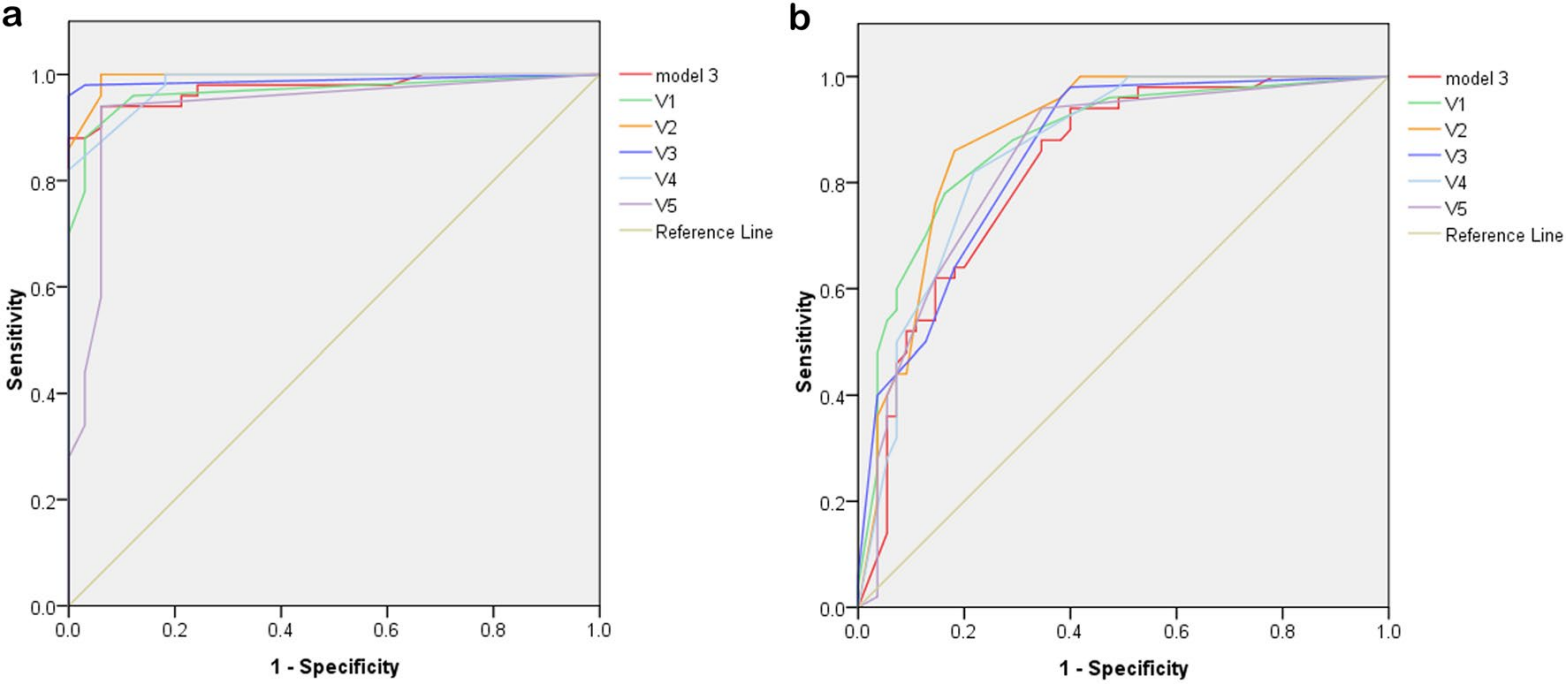
Receiver operating characteristic curves of model 3 and 5 pulmonologists evaluating validation dataset 2. (**a**) TB vs normal, (**b**) TB vs. normal and abnormal other than TB. *TB* tuberculosis. V1–V5 represents the 5 pulmonologists.

**Figure 4:**
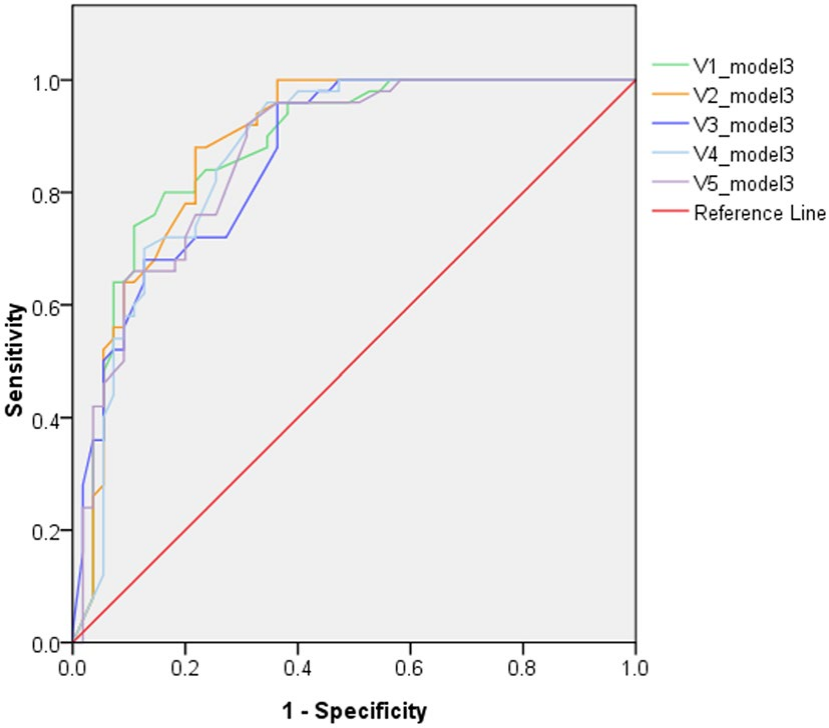
Receiver operating characteristic curves of the 5 pulmonologists that combined with model 3, evaluating validation dataset 2 (TB vs. normal and abnormal other than TB). *TB* tuberculosis. V1–V5 represents the 5 pulmonologists.

Five pulmonologists independently assessed validation dataset 2. The detailed results of the accuracy analysis are presented in Table 4 and Fig. 3a, b. For the analysis of TB vs. normal, the AUC ranged from 0.936 to 0.995. For TB vs. normal and abnormal other than TB, the AUC ranged from 0.843 to 0.888. The AUC of model 3 is close but mild inferior to the five pulmonologists. The overall accuracy and F1 score of Model 3 are similar or even better than pulmonologist. Model 3 has a higher sensitivity than pulmonologists (0.86 vs. 0.34–0.76), while the specificity is lower (0.65–1.0 vs. 0.85–1.0). When combining pulmonologists with model 3 by averaging predictive values, 4 of 5 pulmonologists showed improving of AUC (0.862–0.885, Table 4 and Fig. 4). The radiographic report for validation dataset 2 revealed an even lower sensitivity for TB (0.3), and a good specificity (0.98–1.0).

**Table 4:** Comparison of accuracy between algorithm, pulmonologists and radiology reports in validation dataset 2.

	AUC	Sen	Sp	PPV	NPV	LR+	LR−	OA	F1 score
Validation dataset 2 (TB vs. normal)									
Model 3	0.975	0.86	1.0	1.0	0.82	Infinity	0.14	0.91	0.92
V1	0.967	0.60	1.0	1.0	0.62	Infinity	0.40	0.75	0.75
V2	0.995	0.76	1.0	1.0	0.73	Infinity	0.24	0.85	0.86
V3	0.989	0.50	1.0	1.0	0.56	Infinity	0.50	0.69	0.66
V4	0.982	0.62	1.0	1.0	0.63	Infinity	0.38	0.77	0.76
V5	0.936	0.34	0.97	0.94	0.49	11.3	0.68	0.59	0.50
Radiology report^a^	NA	0.30	1.0	1.0	0.48	Infinity	0.70	0.57	0.46
Validation dataset 2 (TB vs. normal and other abnormality)									
Model 3	0.828	0.86	0.65	0.69	0.83	2.45	0.21	0.75	0.76
V1	0.877	0.60	0.92	0.87	0.71	7.50	0.43	0.76	0.71
V2	0.888	0.76	0.85	0.82	0.79	5.06	0.28	0.80	0.79
V3	0.852	0.50	0.87	0.77	0.65	3.84	0.57	0.69	0.60
V4	0.861	0.62	0.85	0.79	0.71	4.13	0.44	0.74	0.69
V5	0.843	0.34	0.94	0.83	0.61	5.66	0.70	0.65	0.48
Radiology report^a^	NA	0.30	0.98	0.93	0.60	15.0	0.71	0.65	0.45
V1 + model 3	0.880	0.70	0.89	0.85	0.77	6.36	0.34	0.80	0.77
V2 + model 3	0.885	0.72	0.84	0.80	0.77	4.50	0.33	0.78	0.76
V3 + model 3	0.862	0.68	0.82	0.77	0.74	3.78	0.39	0.75	0.72
V4 + model 3	0.869	0.70	0.87	0.83	0.76	5.39	0.35	0.79	0.76
V5 + model 3	0.868	0.64	0.91	0.87	0.74	7.11	0.40	0.78	0.74

V1–V5 represents the 5 pulmonologists.

*AUC *area under curve, *Sen* sensitivity, *Sp* specificity.

^a^Radiology report suspect TB or mycobacterial infection.

Table S2 showed the decisions of pulmonologists on TB sputum exams in each subgroup. The average TB sputum exam rate is 97% in CXR typical TB pattern, 62% in those with abnormal pattern (less like TB). The average TB sputum coverage rate of TB cases is 87%, abnormal other than TB cases is 56%, and normal cases is 2%.

### CXR image patterns and cutoff value evaluation

According to the average result of the five pulmonologists’ interpretation, the CXR image patterns are classified as three categories: typical TB pattern, abnormal pattern (less like TB), and normal pattern. The summary of predictive values of AI models and pulmonologists in each CXR image pattern are provided in Table 5. For model 3, the median predictive value is 0.97 (IQR: 0.64–0.99) in typical TB pattern, 0.5 (IQR: 0.5–0.9) in abnormal pattern (less like TB), and 0.03 (IQR: 0.005–0.13) in normal pattern. The boxplot for distribution of predictive values of model 3 and pulmonologists was shown in Figure S4. A cross table analyzing CXR patterns and disease groups of validation dataset 2 is provided in Table S3, showing that only 26 of 50 TB cases (52%) had typical TB pattern. Meaning while, 4 of 22 of abnormal other than TB cases (18%) presented with CXR pattern mimicking TB. Figure S5 compared the predictive value of model 3 between each disease group and image pattern subgroup. Model 3 had higher predictive values in CXR with typical TB pattern than abnormal pattern (both for TB group and abnormal other than TB group).

**Table 5:** Distributions of predictive values of algorithms and pulmonologists in each image pattern.

CXR patterns	Favor TB pattern (n = 30)	Abnormal pattern (n = 43)	Normal pattern (n = 32)	*p* value
Model 1	1.0 (0.87–1.0)	0 (0–0.89)	0 (0–0.03)	< 0.001
Model 2	0.97 (0.81–1.0)	1.0 (0.98–1.0)	0.01 (0–0.13)	< 0.001
Model 3	0.97 (0.64–0.99)	0.5 (0.5–0.9)	0.03 (0.005–0.13)	< 0.001
V1	0.8 (0.8–0.9)	0.2 (0.1–0.4)	0 (0–0)	< 0.001
V2	0.8 (0.65–0.9)	0.4 (0.3–0.5)	0 (0–0)	< 0.001
V3	0.8 (0.6–0.8)	0.2 (0.2–0.4)	0 (0–0)	< 0.001
V4	0.9 (0.7–0.9)	0.3 (0.1–0.3)	0 (0–0)	< 0.001
V5	0.6 (0.2–0.7)	0.1 (0.1–0.15)	0.05(0.05–0.05)	< 0.001

Data is presented as median (IQR). V1–V5 represents the 5 pulmonologists.

Cutoff value evaluation for model 3 is shown in Table S4. At cutoff value of 0.4, the sensitivity approached 0.92 and 0.94 in validation dataset 1 and 2, respectively. While at cutoff value of 0.8, the specificity is 0.81 and 0.89. When setting sensitivity at 0.90, the specificity is 0.48 and 0.60 in validation dataset 1 and 2, respectively.

### Deployment of the TB CXR AI

Based on the results of this study, we deployed model 3, which had the best accuracy performance, as a readily accessible web application (utilizing JavaScript and TensorFlow.js). This TB CXR AI algorithm can run on a web browser and process data on your device, without sending image to the server. The AI algorithm can be accessed via the following URL: https://www.cxrai-prediction.net/, and the CXR interpretation examples were shown in Figs. S6 and S7. We also provided some examples of TB cases detected by AI algorithm but miss diagnosed by physicians in Fig. S8, and some examples that AI algorithm failed to detect TB in Fig. S9.

## Discussion

In this study, the TB CXR AI algorithm training via Google Teachable Machine with a relatively small number of images, has achieved an acceptable accuracy close to that of professional pulmonologists, and it has a higher sensitivity in TB detection, showing a potential to aid both specialist and non-specialist physicians in enhancing their TB screening sensitivity.

The TB cases collected in this study had relatively high percentage (48%) of atypical CXR pattern. This may be due to older age of our patient group (average 72.7 years old in TB patients). Literature also showed that the percentage of typical TB CXR pattern (upper lung predominant) is significantly influenced by patient’s performance status (PS)^25^. For TB patients with good physical activity (PS of 0), a typical CXR pattern was observed in 71% of cases. As the patient’s physical activity got worse, the proportion of typical CXR patterns drops dramatically (PS = 1: 44%, PS = 2: 19%, PS = 3: 16%, PS = 4: 0%)^25^.

Among the AI models established in this study, model 1 had good specificity but lower sensitivity for TB. However, we found this model was not effective to detect TB with atypical CXR patterns. As for model 2, it is effective to differentiate abnormal CXR from normal cases. Model 3 is the combination of model 1 and 2, and give the average predictive values of the 2 models. This ensemble method can balance the detection of typical and atypical TB, and compensate the occasional false positives and false negatives from model 1 and 2. In theory, typical TB cases would have predictive values near 1 for both model 1 and model 2, averaging around 1. For abnormal cases without a typical TB pattern, model 1 might predict values close to 0, while model 2 would remain near 1, with an average of 0.5. In normal cases, both models would predict values close to 0, resulting in an average also near 0. As evidenced by validation datasets 1 and 2, model 3 successfully achieved the best AUC, which is close to clinical experts.

Both model 3 and the pulmonologists demonstrate excellent accuracy when evaluating TB vs normal. However, when adding abnormal other than TB (mostly pneumonia), the accuracy decreased remarkably in both model 3 (AUC: 0.975 decrease to 0.828) and pulmonologists (AUC: 0.936–0.995 decrease to 0.843–0.888). Pneumonia and other diseases (e.g. pulmonary fibrosis) may also mimic TB. As pneumonia cases increase, the false positives also increase, and we suggest this is the limitation of CXR TB detection, both for human and AI models. However, our study showed that the integration of AI model with physicians’ clinical judgment could potentially improve the overall accuracy of TB detection.

In terms of the performance of pulmonologists and radiology reports, direct comparisons between them maybe not feasible. Because the pulmonologists are already aware that the study is evaluating TB CXRs, and during the exam, the judgment is made under heightened awareness. Therefore, the sensitivity for TB is better than in real-world clinical practice. In contrast, radiology reports are collected retrospectively, reflecting the radiologists’ daily practice at that time. Awareness of TB in these reports is likely lower. On the other hand, the accuracy difference between the radiology reports (sensitivity: 0.3, overall accuracy: 0.65) and the pulmonologists (sensitivity: 0.34–0.76, overall accuracy: 0.65–0.80) also indicates that increasing physicians' awareness of TB may enhance the accuracy of TB CXR evaluations. In this study, pulmonologists tended to perform more extensive TB sputum examinations (even without clinical information), which reflect the experts’ alertness to improve TB detection (70%-98% exam rate in TB cases). Besides, we suggest TB CXR AI may well potentially improve TB awareness for both specialist and non-specialist physicians.

Although in this study, our model showed a lower accuracy than the 5 commercial TB AI algorithms (specificity 48–60% vs. 61–74%, when sensitivity was fixed at 90%)^14^. However, the TB patients in our study are much older (median age 74 vs. 37 years), and the percentage of typical TB image pattern is lower (52%). This difference may decrease the accuracy of AI model in our study. In fact, previous literature also showed decreased accuracy performance of the 5 commercial TB AI algorithms in older age group (> 60 years, AUC range: 0.805–0.864)^14^. This result is getting close to the accuracy of our model (AUC = 0.828) and the pulmonologists (AUC range: 0.843–0.888) in validation dataset 2.

Recent literature has also discussed the problems about TB CXR AI^26^, including the heterogeneity of accuracy across different populations, determination of prediction value thresholds and their variability, and misjudgments in non-TB patients. Therefore, this study used actual clinical CXRs for external validation to confirm accuracy in clinical situation. The determination of thresholds is both a strength and limitation of AI models. Therefore, this study also conducted a cutoff value evaluation to help determine the relationship of predictive value and accuracy.

The limitations of this study were as follows. First, the image recognition of GoogleTM operates on a relatively small resolution (224 × 224 pixels). Therefore, this AI algorithm can only identify large and obvious image features, and small lung lesions may be missed. Second, the AI model used in this study could not locate lesions. Third, this is a single center retrospective study, and the size of the validation dataset is relatively small. The accuracy result may not be generalizable to different CXR machine and settings. Fourth, this AI model is not optimal for detect TB cases without a typical TB pattern. However, physicians also have the similar limitation. Fifth, we did not evaluate the accuracy of radiologists. However, the retrospectively collected radiology reports may reflect the accuracy of daily clinical practice of radiologists. Finally, we did not evaluate the accuracy of frontline medical staffs such as junior residents and nurse practitioners. However, we can expect their accuracy for TB detection would be lower than expert physicians. And AI algorithm may be more helpful for them.

In conclusion, this study developed an open and free AI algorithm, which is effective in detection of typical TB features on CXR. The accuracy is acceptable and may be close to the clinical experts. We suggest a predictive value > 0.9 for high TB probability. For predictive value 0.5–0.9, abnormal pattern is favored, and TB may be considered. For predictive value < 0.4, TB is unlikely. Further research with larger scale validation to evaluate the generalizability of the algorithm, and compare the performance in different population, is required.

### Supplementary Information

Supplementary Information.

## Data Availability

The datasets used and/or analyzed during the current study are available from the corresponding author on reasonable request.
