# Supplementary material for: A deep learning-based algorithm for pulmonary tuberculosis detection in chest radiography
Source: Sci Rep. 2024 Jun 28;14:14917. doi: 10.1038/s41598-024-65703-z (PMC11213931; doi:10.1038/s41598-024-65703-z)

**Supplementary Materials**

Table S1. Confusion matrix of AI models.

|  |  |  | predict | |  |  | predict | |  |  |  |  |
| --- | --- | --- | --- | --- | --- | --- | --- | --- | --- | --- | --- | --- |
| **Internal validation** |  | **model 1** | TB | Normal |  | **model 2** | Abnormal | Normal |  |  |  |  |
|  | class | TB | 51 | 2 | class | Abnormal | 354 | 30 |  |  |  |  |
|  |  | Normal | 14 | 557 |  | Normal | 15 | 174 |  |  |  |  |
|  |  |  |  |  |  |  |  |  |  |  |  |  |
|  |  |  | predict | |  |  | predict | |  |  | predict | |
| **External validation 1** |  | **model 1** | TB | Normal |  | **model 2** | Abnormal | Normal |  | **model 3** | TB | Not TB |
|  | class | TB | 54 | 29 | class | TB | 69 | 14 | class | TB | 73 | 10 |
|  |  | Normal | 9 | 75 |  | Normal | 14 | 70 |  | Normal | 4 | 80 |
|  |  | Abnormal^*^ | 36 | 47 |  | Abnormal^*^ | 78 | 5 |  | Abnormal^*^ | 76 | 7 |
|  |  |  |  |  |  |  |  |  |  |  |  |  |
|  |  |  | predict | |  |  | predict | |  |  | predict | |
| **External validation 2** |  | **model 1** | TB | Normal |  | **model 2** | Abnormal | Normal |  | **model 3** | TB | Not TB |
|  | class | TB | 33 | 17 | class | TB | 42 | 8 | class | TB | 43 | 7 |
|  |  | Normal | 2 | 31 |  | Normal | 3 | 30 |  | Normal | 0 | 33 |
|  |  | Abnormal^*^ | 6 | 16 |  | Abnormal^*^ | 20 | 2 |  | Abnormal^*^ | 19 | 3 |

^*^Abnormal other than TB

Table S2. The summary of interpretation results of pulmonologists and their decisions on TB sputum exams for each subgroup.

| Validation dataset 2 | V1 | V2 | V3 | V4 | V5 | V1-V5 average |
| --- | --- | --- | --- | --- | --- | --- |
| Typical TB pattern, n(%) | 32 (30) | 30 (28) | 28 (26) | 26 (24) | 19 (18) | 27 (26) |
| Abnormal pattern (less like TB) , n(%) | 36 (34) | 46 (44) | 45 (43) | 55 (52) | 55 (52) | 47.4 (45) |
| Normal pattern, n(%) | 37 (35) | 29 (28) | 32 (30) | 24 (23) | 31 (30) | 30.6 (29) |
|  |  |  |  |  |  |  |
| Check TB sputum, n(%) | 41 (39) | 56 (53) | 70 (66) | 61 (58) | 56 (53) | 56.8 (54) |
| Sputum TB exam rate in image pattern subgroup, n(%) | | | | | | |
| Typical TB pattern | (93) | (100) | (100) | (96) | (100) | (97) |
| Abnormal pattern (less like TB) | (30) | (58) | (93) | (74) | (55) | (62) |
| Sputum TB exam rate in disease subgroup, n(%) | | | | | | |
| TB case | (70) | (88) | (98) | (94) | (86) | (87) |
| Abnormality other than TB | (27) | (50) | (90) | (63) | (50) | (56) |
| Normal case | (0) | (3) | (3) | (0) | (6) | (2) |

Data is presented as n (%). V1-V5 represents the 5 pulmonologists.

Table S3. Cross table for relationship of disease groups and image patterns.(Validation dataset 2)

|  |  | Image pattern group | |  |
| --- | --- | --- | --- | --- |
|  |  | TB pattern | Abnormal pattern (less like TB) | Normal pattern |
| Disease group | TB | 26 | 24 | 0 |
|  | Abnormal other than TB | 4 | 18 | 0 |
|  | Normal | 0 | 1 | 32 |

Table S4. Cutoff value evaluation for model 3 using validation dataset 1.

|  | Cutoff level | Sen | Sp | PPV | NPV | LR+ | LR- | Overall accuracy | F1 score | |
| --- | --- | --- | --- | --- | --- | --- | --- | --- | --- | --- |
| Validation dataset 1 (TB vs. normal and other abnormality) | | | | | | | | | |  |
| model 3 | 0.1 | 0.95 | 0.31 | 0.40 | 0.92 | 1.37 | 0.16 | 0.52 | 0.56 | |
|  | 0.2 | 0.94 | 0.37 | 0.42 | 0.92 | 1.49 | 0.16 | 0.55 | 0.58 | |
|  | 0.3 | 0.92 | 0.40 | 0.43 | 0.91 | 1.53 | 0.20 | 0.57 | 0.58 | |
|  | 0.4 | 0.92 | 0.44 | 0.44 | 0.91 | 1.64 | 0.18 | 0.59 | 0.60 | |
|  | 0.5 | 0.88 | 0.52 | 0.47 | 0.89 | 1.83 | 0.23 | 0.64 | 0.61 | |
|  | 0.6 | 0.60 | 0.76 | 0.55 | 0.79 | 2.50 | 0.52 | 0.70 | 0.57 | |
|  | 0.7 | 0.57 | 0.78 | 0.56 | 0.78 | 2.59 | 0.55 | 0.71 | 0.56 | |
|  | 0.8 | 0.49 | 0.81 | 0.56 | 0.76 | 2.57 | 0.63 | 0.70 | 0.52 | |
|  | 0.9 | 0.42 | 0.85 | 0.58 | 0.74 | 2.80 | 0.68 | 0.70 | 0.48 | |
|  | 0.99 | 0.26 | 0.91 | 0.58 | 0.71 | 2.88 | 0.81 | 0.69 | 0.36 | |
| Validation dataset 2 (TB vs. normal and other abnormality) | | | | | | | | | | |
| model 3 | 0.2 | 0.96 | 0.50 | 0.63 | 0.93 | 1.92 | 0.08 | 0.71 | 0.76 | |
|  | 0.4 | 0.94 | 0.56 | 0.66 | 0.91 | 2.13 | 0.10 | 0.74 | 0.77 | |
|  | 0.5 | 0.86 | 0.65 | 0.69 | 0.83 | 2.45 | 0.21 | 0.75 | 0.76 | |
|  | 0.6 | 0.62 | 0.83 | 0.76 | 0.70 | 3.64 | 0.45 | 0.73 | 0.68 | |
|  | 0.7 | 0.60 | 0.85 | 0.78 | 0.70 | 4.00 | 0.47 | 0.73 | 0.68 | |
|  | 0.8 | 0.54 | 0.89 | 0.81 | 0.68 | 4.90 | 0.51 | 0.72 | 0.65 | |
|  | 0.9 | 0.46 | 0.92 | 0.83 | 0.65 | 5.75 | 0.58 | 0.70 | 0.59 | |

LR = likelihood ratio, NPV = negative predictive value ; PPV = positive predictive value; Sen = sensitivity; Sp = specificity.

Figure S1. Accuracy curve and loss function of model 1. The hyperparameters are learning rate = 0.001, batch size = 64, and epochs = 50.


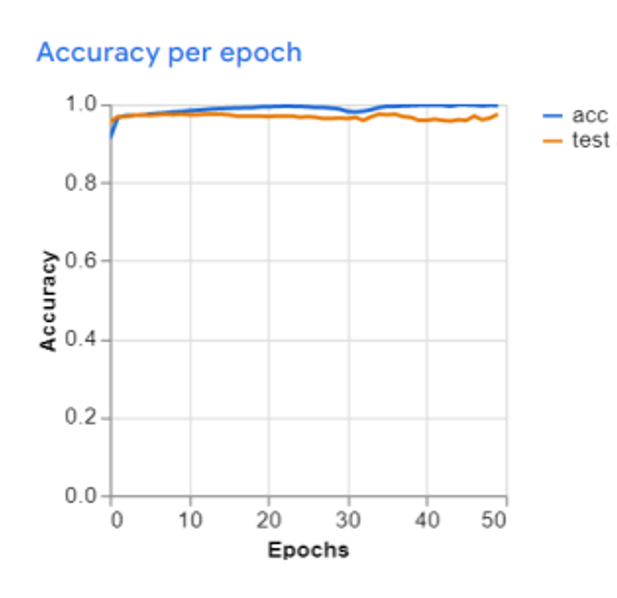

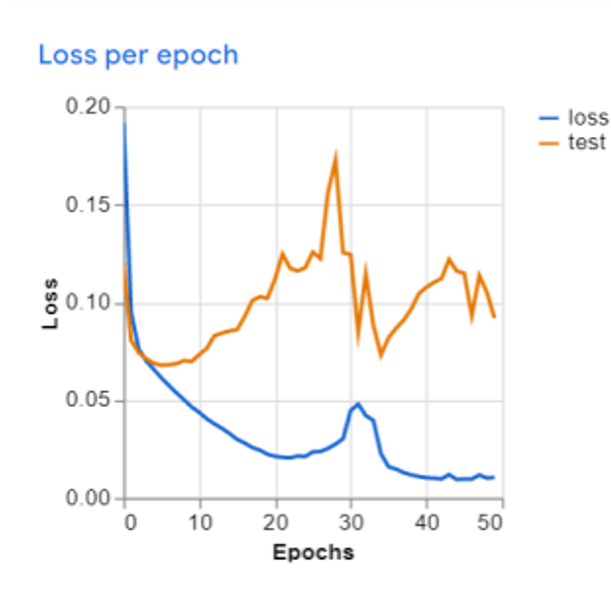


Figure S2. Accuracy curve and loss function of model 2. The hyperparameters are learning rate = 0.001, batch size = 128, and epochs = 50.


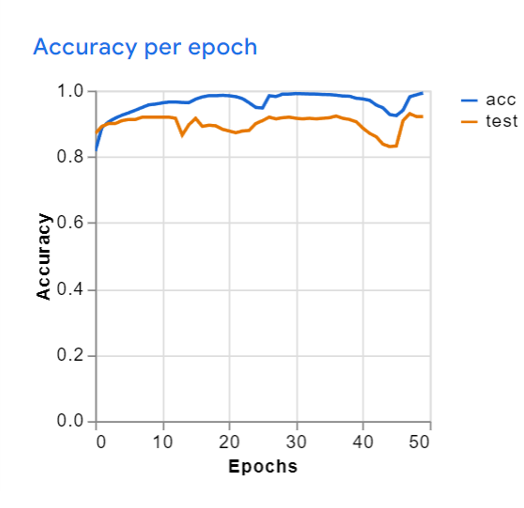

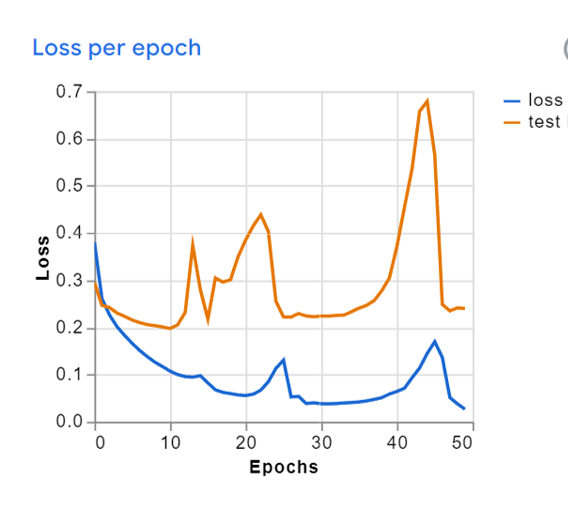


Figure S3. Figure S3. The distribution of predictive values of the models in each disease subgroup. (Validation dataset 1)


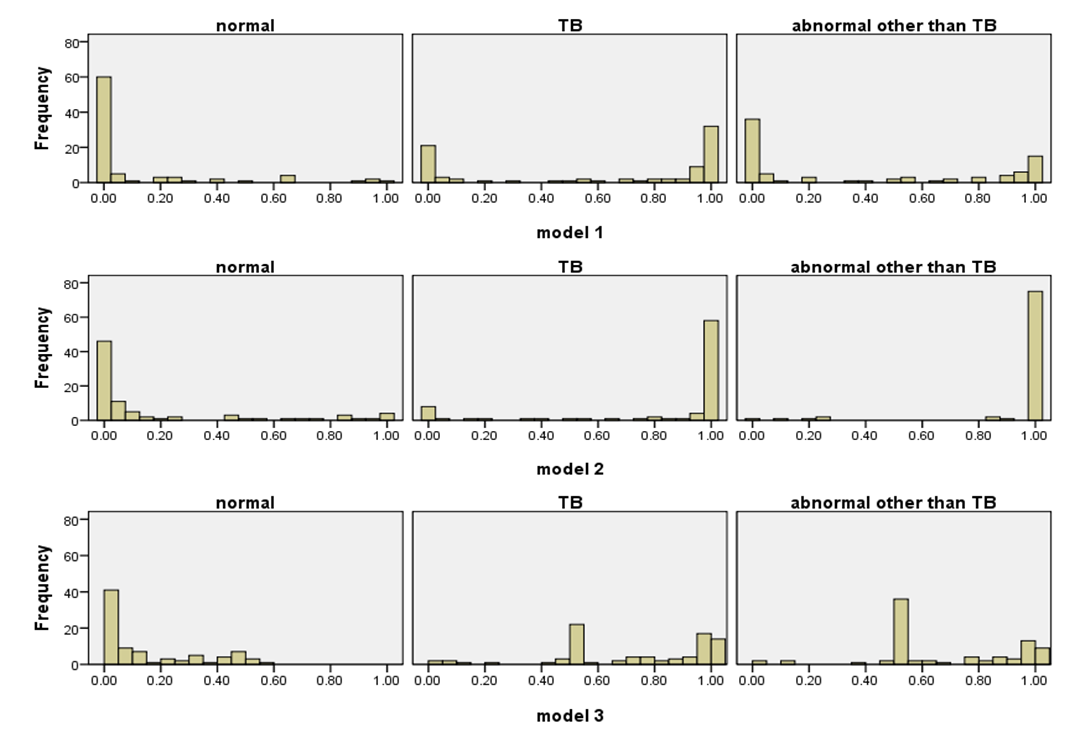


Figure S4. The distribution of predictive values of model 3 and pulmonologists in each image pattern subgroup. (TB pattern vs. abnormal pattern less like TB vs. normal pattern. Validation dataset 2) V1-V5 represents the 5 pulmonologists.


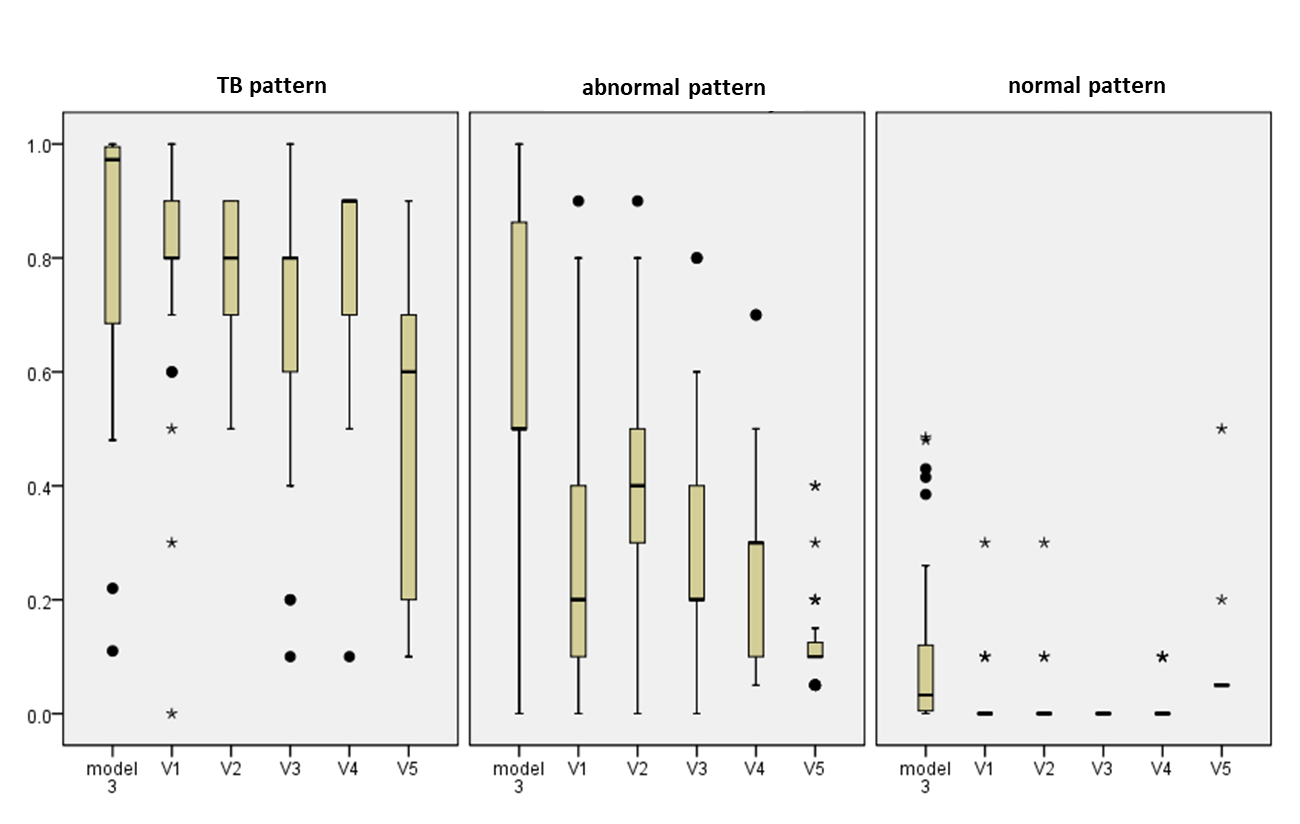


Figure S5. The comparison of predictive values of model 3 in each disease group and image pattern subgroup. (Validation dataset 2)


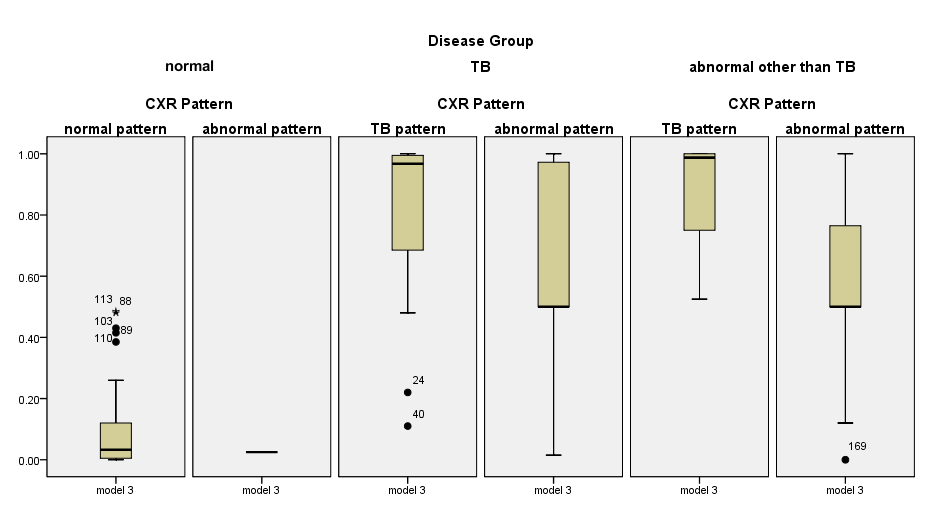


Figure S6. Interpretation examples of TB CXRs by the web-based TB CXR AI algorithm. <https://www.cxrai-prediction.net/>


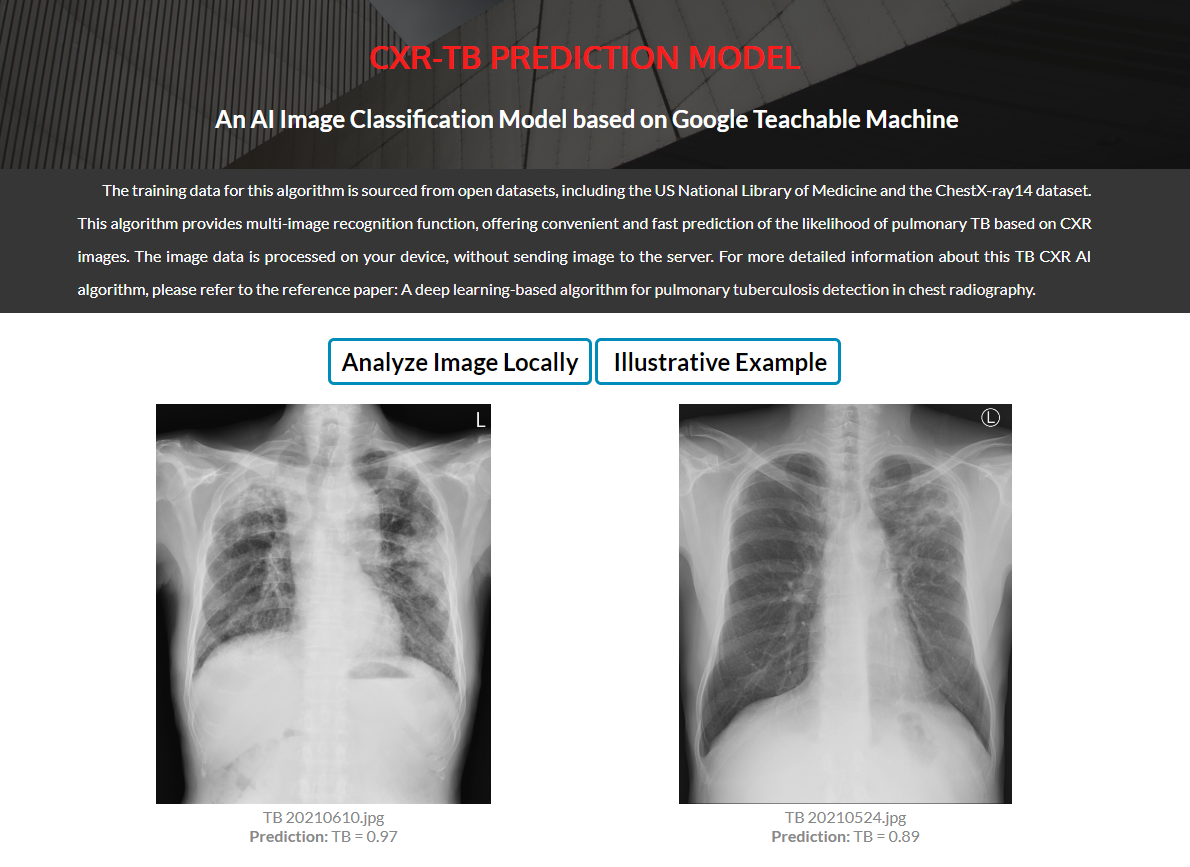


Figure S7. Interpretation examples of pneumonia and normal CXRs by the web-based TB CXR AI algorithm.


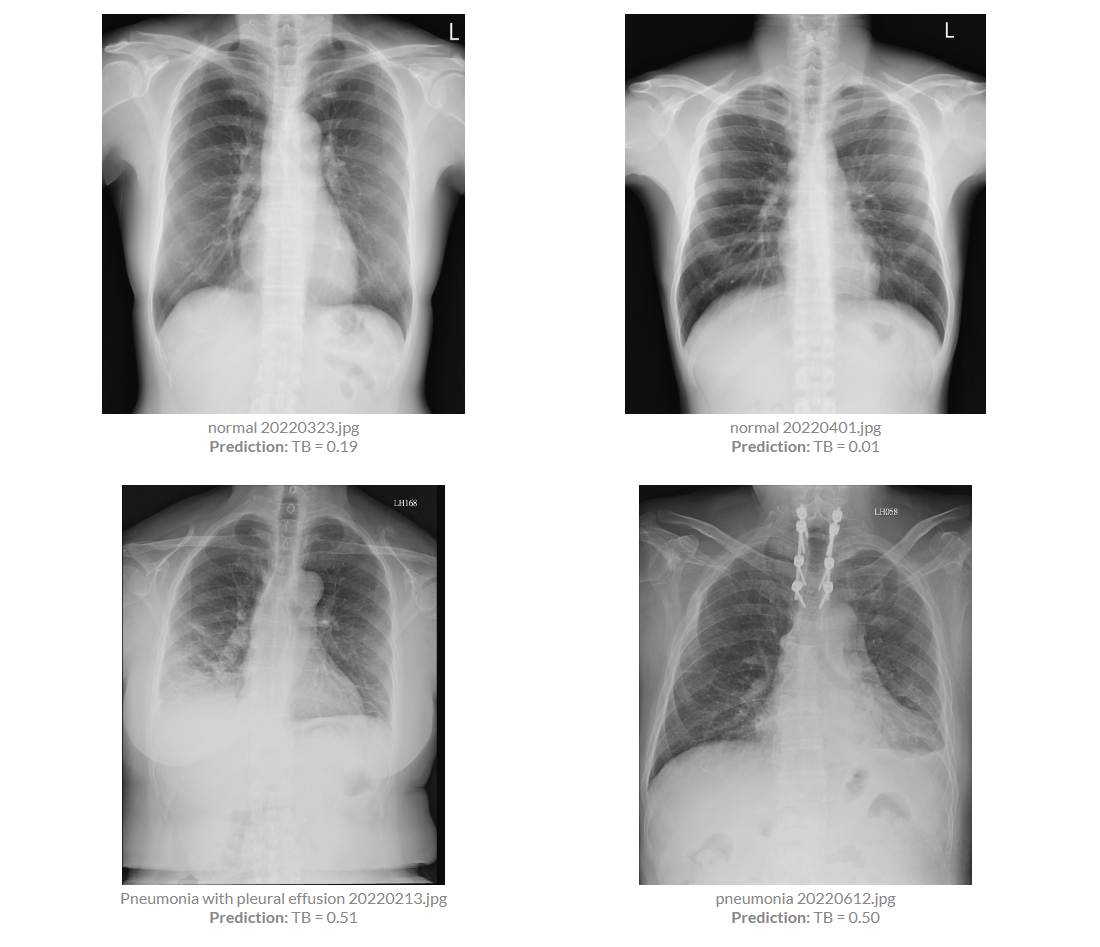


Figure S8. Interpretation examples of TB detected by AI algorithm but missed by physicians. Left: physician prediction = 0.2-0.5. Right: physician prediction = 0.1-0.5.


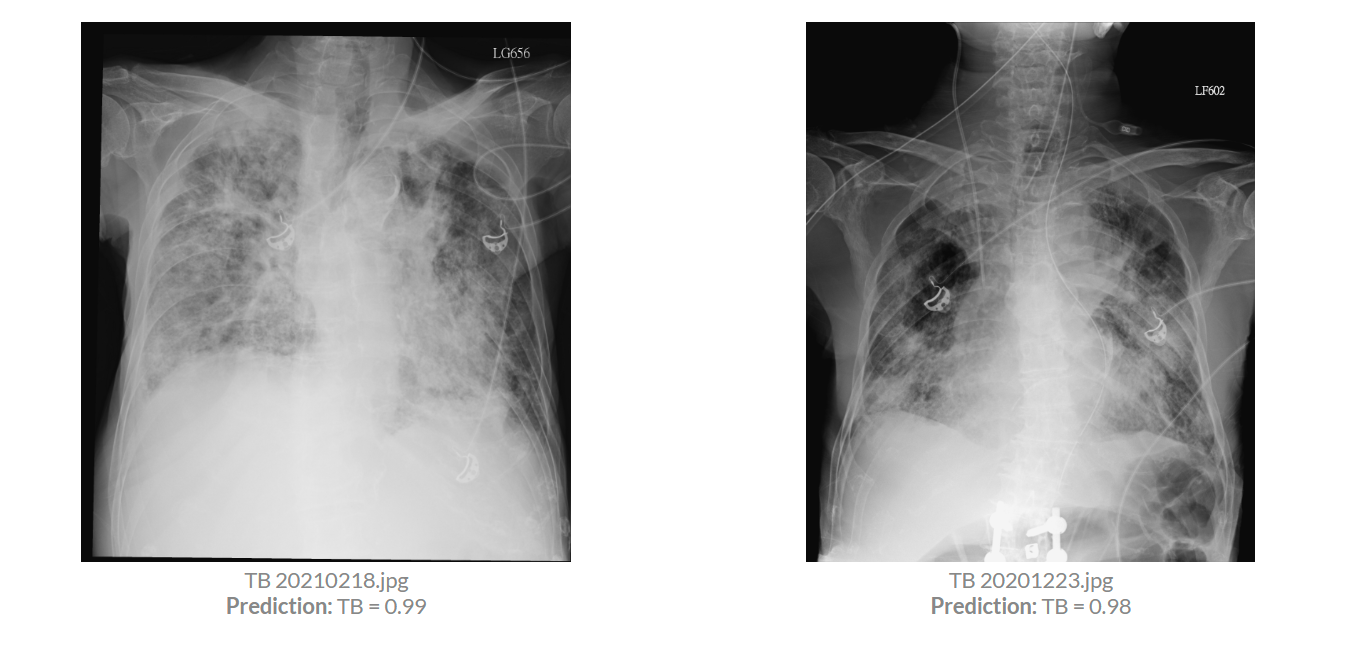


Figure S9. Interpretation examples of TB detected by physicians, but AI algorithm predictive values were low and not helpful. Left: physician prediction = 0.8-0.9. Right: physician prediction = 0.5-1.0.


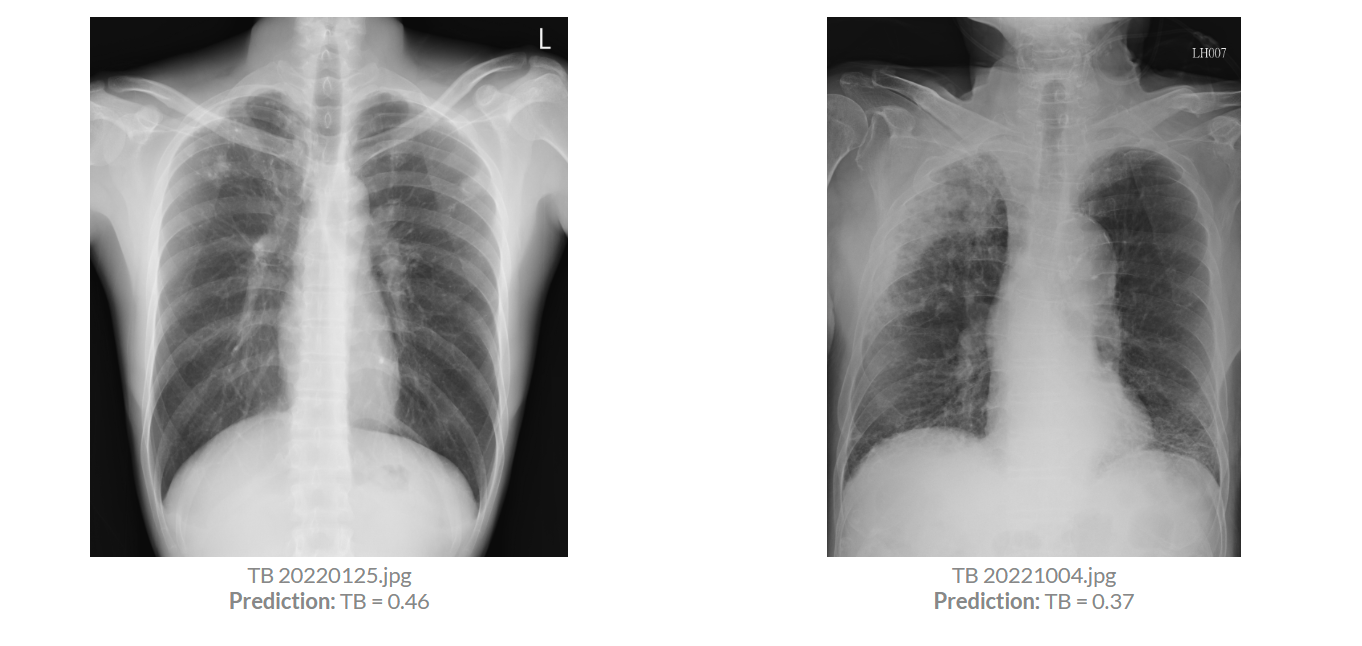

Supplement: Supplementary file 1 — Supplementary Information. [file 41598_2024_65703_MOESM1_ESM.docx]
